# PinX1 inhibits the invasion and metastasis of human breast cancer via suppressing NF-κB/MMP-9 signaling pathway

**DOI:** 10.1186/s12943-015-0332-2

**Published:** 2015-03-26

**Authors:** Meilin Shi, Menghan Cao, Jun Song, Qinghua Liu, Hailong Li, Fei Meng, Zhenqiang Pan, Jin Bai, Junnian Zheng

**Affiliations:** Jiangsu Center for the Collaboration and Innovation of Cancer Biotherapy, Cancer Institute, Xuzhou Medical College, 84 West Huaihai Road, Xuzhou, 221002 Jiangsu Province China; School of Medical Imaging, Xuzhou Medical College, Xuzhou, Jiangsu China; Department of Pathology, Xuzhou Medical College, Xuzhou, Jiangsu China; Department of Urology, the Affiliated Hospital of Xuzhou Medical College, Xuzhou, Jiangsu China; Department of Oncological Sciences, Icahn School of Medicine at Mount Sinai, New York, NY USA

**Keywords:** PinX1, Breast cancer, Metastasis, Prognostic, NF-κB, MMP-9

## Abstract

**Background:**

PinX1 (PIN2/TRF1-interacting telomerase inhibitor 1) was suggested to be correlated with tumor progression. This study was designed to evaluate the role of PinX1 in human breast cancer.

**Methods:**

To evaluate the function of PinX1 in breast cancer, we used a tissue microarray (TMA) of 405 human breast cancer patients and immunohistochemistry to analyze the correlation between PinX1 expression and clinicopathologic variables and patient survival. We also detected the abilities of cell migration and invasion in breast cancer by performing cell migration and invasion assay, gelatin zymography and western blot analysis. Lastly, we set up the nude mice model by Tail vein assay to exam the functional role of PinX1 in breast cancer metastasis.

**Results:**

We found that low PinX1 expression was associated with lymph node metastasis (*P* = 0.002) and histology grade (*P* = 0.001) in patients, as well as with poorer overall and disease-specific survival (*P* = 0.010 and *P* = 0.003, respectively). Moreover, we identified that PinX1 inhibited the migration and invasion of breast cancer by suppressing MMP-9 expression and activity via NF-κB-dependent transcription *in vitro*. Finally, our mice model confirmed that PinX1 suppressed breast cancer metastasis *in vivo*.

**Conclusions:**

Our data revealed that low PinX1 expression was an independent negative prognostic factor for breast cancer patients. These findings suggested that PinX1 might be function as a tumor metastasis suppressor in the development and progression of breast cancer by regulating the NF-κB/MMP-9 signaling pathway, and might be a prognostic marker as well as a therapeutic target for breast cancer.

**Electronic supplementary material:**

The online version of this article (doi:10.1186/s12943-015-0332-2) contains supplementary material, which is available to authorized users.

## Introduction

Breast cancer is the most common malignancy of the female, and its survival rate falls from 90% for localized to 20% for metastatic disease [1]. Each year there are approximately 400 000 deaths because of breast cancer [2]. The high mortality is related to complications of tumor dissemination and distant metastasis. Metastasis is a multistep process requiring cell proliferation, migration, invasion, adhesion, vessel formation and colonization to a secondary site [3]. Therefore, interrupting the metastatic process is a key to decreasing breast cancer mortality. Alterations in chromatin play a critical role in breast cancer progression and metastasis, but the exact molecular mechanisms are still limited [4].

Human telomerase is a ribonucleoprotein, mainly consisting of catalytic subunit hTERT and RNA template hTR, which involves in malignant tumor formation [5-7]. Human telomerase reverse transcriptase (hTERT), containing two conserved N-terminal and four C-terminal domains essential for telomerase catalytic activity, is referred as the rate-limiting step of telomerase activation [8]. Telomerase expression is suppressed in most normal cells, whereas reactivated in more than 85% of human cancer cells [9,10]. In addition, the telomerase activity is regulated by telomeric repeat binding factor 1 (TRF1) and its associated proteins, including PinX1 [11]. PinX1 is a potent telomerase inhibitor and a putative tumor suppressor, firstly found as a Pin2/TRF1-binding protein [12]. However, unlike other TRF1-binding proteins, PinX1 is unique in that it can also directly bind to hTERT and inhibit telomerase activity [12].

PinX1 is a versatile gene at human chromosome 8p23, a region frequently associated with loss of heterozygosity (LOH) in a variety of human malignancies [13-15]. The full-length form of PinX1 is composed of 737 bp and encodes a 45-KDa nucleolar protein containing 328 amino acids [16]. It has been identified that Pinx1 deficiency could induce telomerase activation, telomere elongation and chromosome instability [17], whereas overexpression of PinX1 leads to a decrease in both telomerase activity and cancer cell tumorigenicity [18,19]. LOH of PinX1 resulted in gastric carcinoma development, which suggested PinX1 might have a potential inhibitory role in cancer metastasis [20]. Then increasing evidences demonstrate that PinX1 plays a key role as a putative tumor suppressor in human cancer progression [21-26]. However, the PinX1 expression status and its correlation with the clinicopathological features in breast cancer have never been investigated. In addition, the potential molecular mechanisms underlying the role of PinX1 in breast cancer are still unclear.

To evaluate the function of PinX1 in breast cancer, we used a tissue microarray (TMA) of human breast cancer patients and immunohistochemistry to analyze the correlation between PinX1 expression and clinicopathologic variables and patient survival. Furthermore, we demonstrated that PinX1 suppressed breast cancer migration and invasion by inhibiting the expression and activity of MMP-9 via NF-κB-dependent transcription *in vitro* and *in vivo*. These data suggested that PinX1 might be function as a tumor metastasis suppressor in the development and progression of breast cancer by regulating the NF-κB/MMP-9 signaling pathway, and might be a prognostic marker as well as a therapeutic target for breast cancer.

## Results

### Correlation of PinX1 staining with clinicopathologic parameters in breast cancer patients

**Figure 1:**
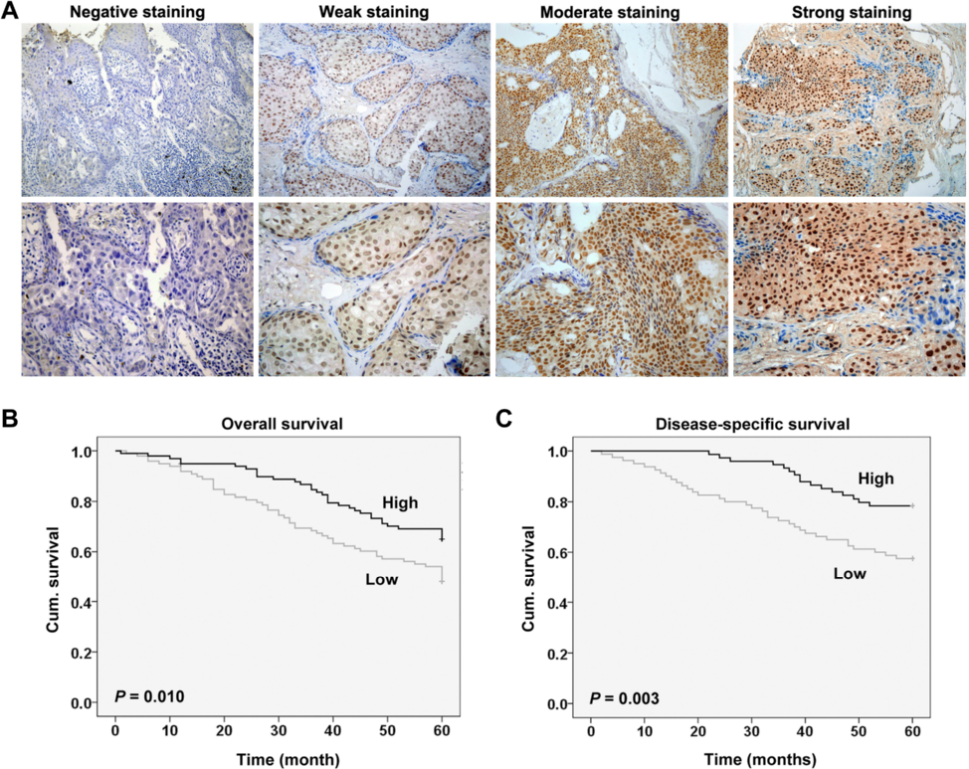
**Expression of PinX1 in breast cancer tissues and associated with 5-year overall and disease-specific survival in breast cancer patients. (A)** Negative and positive staining in breast cancer tissue. Top panel, magnification × 200; bottom panel, magnification × 400. **(B)** Low PinX1 expression correlated with a poorer 5-year overall cumulative survival for 236 breast cancer patients (*P* = 0.010, log-rank test). **(C)** Low PinX1 expression correlated with a poorer 5-year disease-specific cumulative survival for 208 breast cancer patients (*P* = 0.003, log-rank test). Cum. indicates cumulative.

To investigate PinX1 expression in breast cancer, immunohistochemistry was carried out in TMA slides (Figure 1A). Samples with IRS 0–3 and IRS 4–12 were classified as low and high expression of PinX1. Of the 405 breast cancer analyzed, low and high expression of PinX1 staining were 52.3% (212/405) and 47.7% (193/405), respectively (Table 1). We then analyzed the correlations between PinX1 expression and characteristics of the breast carcinomas, and found that PinX1 staining was dramatically decreased in histology grade II and III compared with histology grade I (*P* = 0.001, χ^2^ test, Table 1). We also found that PinX1 expression is significantly correlated with lymph node metastasis (*P* = 0.002, χ^2^ test, Table 1). However, we did not find any significant correlations between PinX1 expression and other clinicopathologic variables, including patient age, tumor size, ER status, PR status, HER2 status and p53 status.

**Table 1:** **PinX1 staining and clinicopathological characteristics of 405 breast cancer patients**

**Variables**	**PinX1 staining**			
	**Low (%)**	**High (%)**	**Total**	***P*** *
**Age**				
≤50 years	99 (52.7)	89 (47.3)	188	0.906
>50 years	113 (52.1)	104 (47.9)	217	
**Tumor size**				
T1 (<2 cm)	47 (61.8)	29 (38.2)	76	0.090
T2 (2-5 cm)	148 (51.2)	141 (48.8)	289	
T3 (>5 cm)	11 (39.3)	17 (60.7)	28	
**Lymph node metastasis**				
Negative	83 (43.5)	108 (56.5)	191	0.002
Positive	110 (59.5)	75 (40.5)	185	
**Histology grade**				
I	11 (30.6)	25 (69.4)	36	0.001
II	103 (50.5)	101 (49.5)	204	
III	50 (68.5)	23 (31.5)	73	
**ER status**				
Negative	40 (54.8)	33 (45.2)	73	0.359
Positive	63 (48.1)	68 (51.9)	131	
**PR status**				
Negative	48 (54.5)	40 (45.5)	88	0.313
Positive	55 (47.4)	61 (52.6)	116	
**HER2 status**				
Negative	14 (66.7)	7 (33.3)	21	0.206
Positive	79 (52.0)	73 (48.0)	152	
**p53 status**				
Negative	41 (50.0)	41 (50.0)	82	0.347
Positive	52 (57.1)	39 (42.9)	91	

^*^ *P* values are from χ^2^ test.

Some cases were not available for the information.

### PinX1 functions as an independent prognostic factor for human breast cancer

To further study whether PinX1 expression correlates with the survival of patients, Kaplan-Meier survival curves were constructed using 5-year overall and disease-specific cumulative survival to compare the patients with high PinX1 staining to those with low PinX1 staining (n = 236, follow-up time, 60 months). Our data revealed that low PinX1 staining correlated with both poorer overall and disease-specific patient survival (*P* = 0.010 and *P* = 0.003, respectively, log-rank test; Figure 1B & C). The 5-year overall cumulative survival rate dropped from 65.3% in patients with high PinX1 expression to 48.0% in those with low PinX1 expression, and the 5-year disease-specific cumulative survival rate dropped from 78.4% in patients with high PinX1 expression to 57.5% in those with low PinX1 expression.

Moreover, we examined whether PinX1 expression was an independent prognostic factor for breast cancer. The univariate Cox regression analyses revealed that PinX1 expression was an independent prognostic marker for breast cancer patients overall survival (hazard ratio, 0.573; 95% CI, 0.371-0.884; *P* = 0.012; Table **2**) and disease-specific survival (hazard ratio, 0.417; 95% CI, 0.230-0.755; *P* = 0.004; Table **2**). In multivariate Cox regression analysis, we found that PinX1 expression was also an independent prognostic marker for both 5-year overall survival (hazard ratio, 0.527; 95% CI, 0.404-0.656; *P* = 0.027; Table 3) and disease-specific survival (hazard ratio, 0.429; 95% CI, 0.222-0.630; *P* = 0.012; Table 3). Our results definitely confirmed that low PinX1 expression is associated with poor prognosis, suggesting that PinX1 may function as a prognostic marker for breast cancer.

**Table 2:** **Univariate Cox proportional regression analysis on 5-year overall and disease-specific survival of 405 breast cancer patients**

**Variable** ^*^	**Overall survival**			**Disease-specific survival**		
	**Hazard ratio**	**95% CI** ^**†**^	***P*** ^*^	**Hazard ratio**	**95% CI** ^**†**^	***P*** ^*^
PinX1						
Low	1.000		0.012	1.000		0.004
High	0.573	0.371-0.884		0.417	0.230-0.755	
Age						
≤50 years	1.000		0.443	1.000		0.630
>50 years	0.864	0.594-1.225		0.855	0.632-1.030	
Tumor size						
≤5 cm	1.000		0.002	1.000		0.000
>5 cm	2.289	1.363-3.843		3.463	2.730-3.935	
Lymph node metastasis						
Negative	1.000		0.000	1.000		0.000
positive	4.994	3.032-8.227		3.564	2.594-4.505	
Histology Grade						
I	1.000		0.002	1.000		0.005
II/III	2.309	1.740-3.456		2.554	1.618-3.551	

^*^ *P* values are from Log-rank test.

^†^ CI: confidence interval.

**Table 3:** **Multivariate Cox regression analysis on 5-year overall and disease-specific survival of 405 breast cancer patients**

**Variable** ^*^	**Overall survival**			**Disease-specific survival**		
	**Hazard ratio**	**95% CI** ^**†**^	***P***	**Hazard ratio**	**95% CI**	***P***
PinX1	0.527	0.404 to 0.656	0.027	0.429	0.222 to 0.630	0.012
Age	0.993	0.740 to 1.345	0.836	0.856	0.652 to 1.222	0.884
Tumor size	2.730	1.947 to 3.785	0.003	2.913	1.882 to 4.146	0.001
Lymph node metastasis	3.183	1.911 to 4.505	0.001	3.962	2.975 to 4.756	0.000
Histology Grade	1.840	1.184 to 2.843	0.023	1.958	1.514 to 2.511	0.032

^*^Coding of variables: PinX1 was coded as 1 (low), and 2 (high). Age was coded as 1 (≤50 years), and 2 (>50 years). Tumor size was coded as 1 (≤5 cm), and 2 (>5 cm). Lymph node metastasis was coded as 1 (negative), and 2 (positive). Histology grade was coded as 1 (I), and 2 (II and III).

^†^ CI: confidence interval.

### PinX1 inhibits migration and invasion of human breast cancer cells in vitro

**Figure 2:**
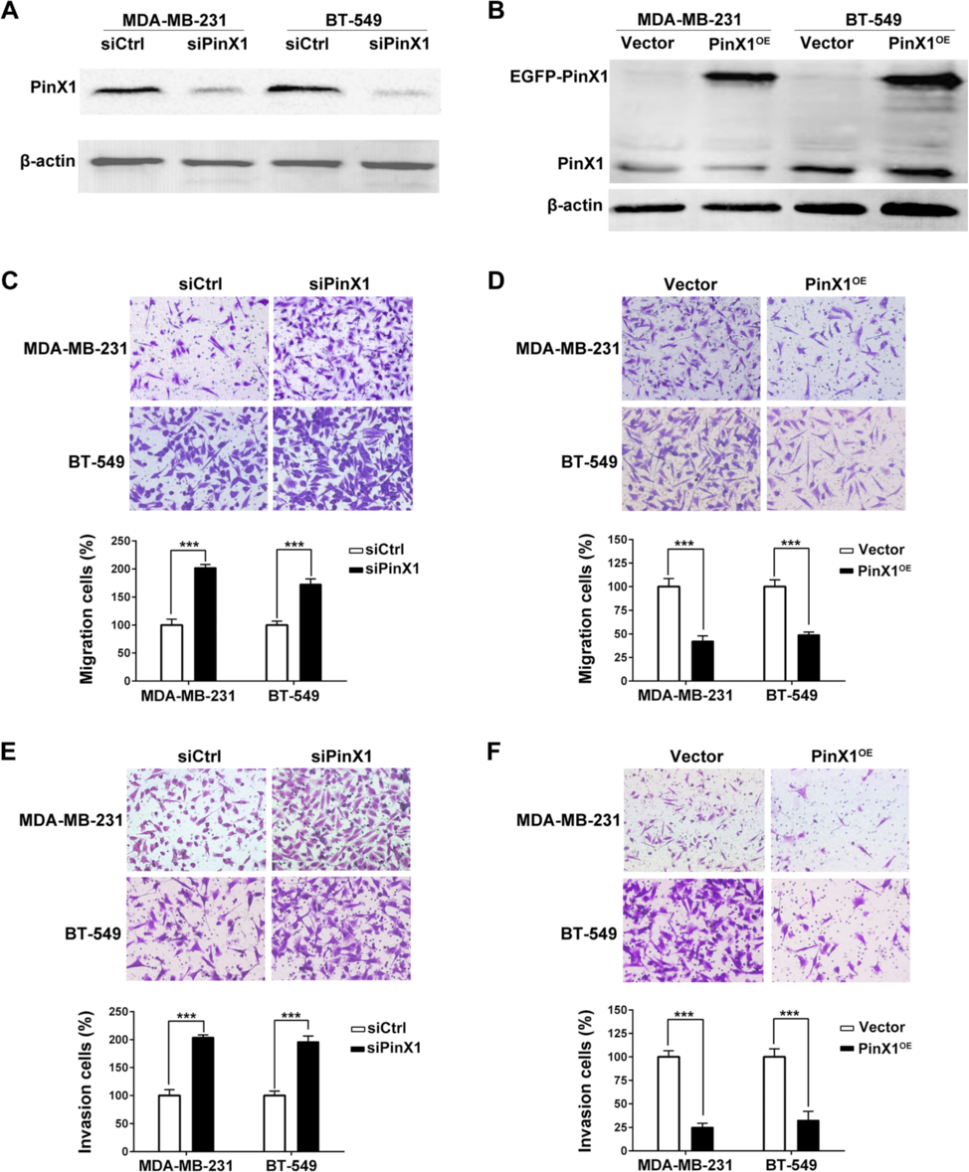
**PinX1 inhibits migration and invasion of human breast cancer cells. (A)** Western blot analysis of the relative protein level of PinX1 in PinX1 knockdown (siPinX1) and control siRNA (siCtrl) groups for both MDA-MB-231 and BT-549 cell lines. **(B)** Western blot analysis of the relative protein level of PinX1 in PinX1 overexpression (PinX1^OE^) and control vector (Vector) groups for both MDA-MB-231 and BT-549 cell lines. **(C)** and **(E)** PinX1 knockdown significantly inhibited migration and invasion abilities of MDA-MB-231 and BT-549 cells. **(D)** and **(F)** PinX1 overexpression significantly inhibited migration and invasion abilities of MDA-MB-231 and BT-549 cells. All experiments were carried out in triplicate. Data are shown as means ± SD. ***, *P* < 0.001.

Because low PinX1 expression is associated with poor prognosis, supporting PinX1 may play important roles in one or more steps of tumor metastasis. We investigated the involvement of PinX1 in breast cancer cells migration and invasion. We transiently transfected MDA-MB-231 and BT-549 cells with control siRNA and PinX1 siRNA or pEGFP-C3 and pEGFP-C3-PinX1 plasmids, respectively. Forty-eight or twenty-four hours after transfection, PinX1 protein was significantly knockdown or overexpressed in cancer cells, respectively (Figure 2A & B). In cell migration assay, we found that the ability of cell migration was drastically increased after PinX1 knockdown in both MDA-MB-231 and BT-549 cell line (Figure 2C). In contrast, overexpression of PinX1 inhibited cell migration (Figure 2D). Meanwhile, the results of the cell invasion assay corresponded with the cell migration assay (Figure 2E & F). However, overexpression or knockdown of PinX1 had no detectable effect on the proliferation of breast cancer cells under normal culture conditions (data not shown).

### PinX1 inhibits human breast cancer cells’ migration and invasion abilities by suppressing MMP-9 expression and activity

**Figure 3:**
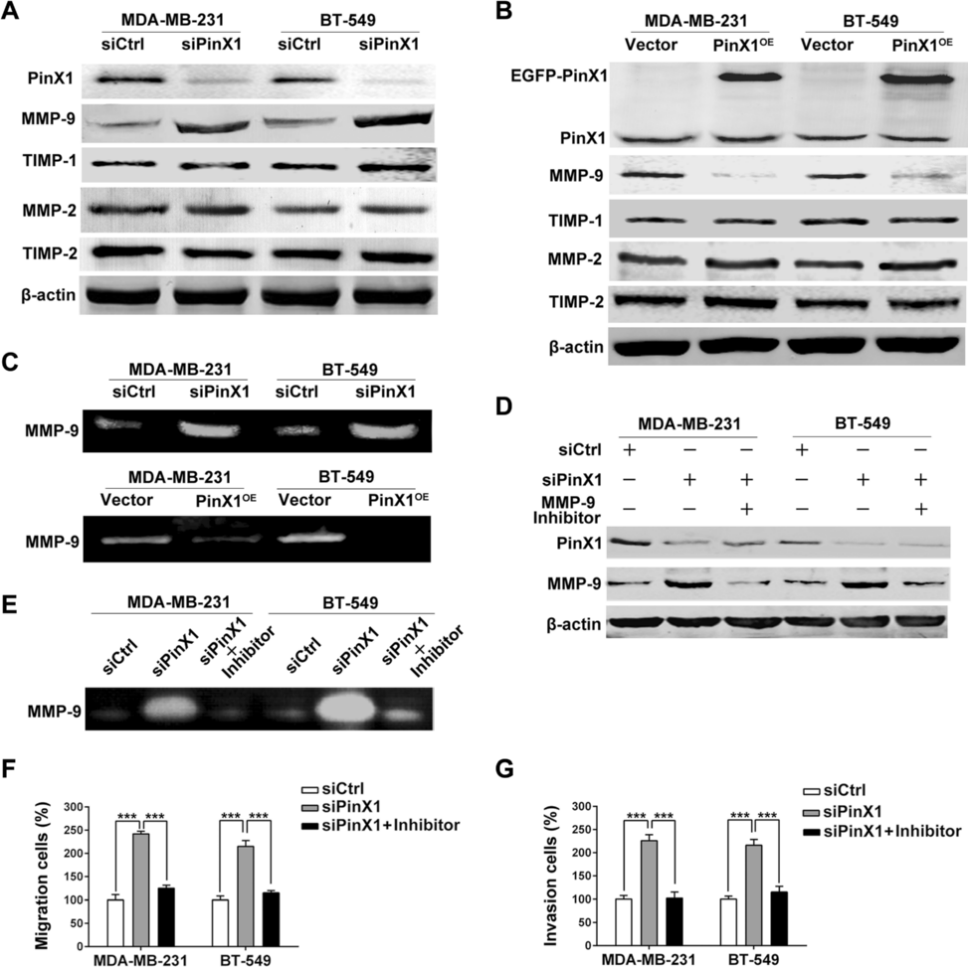
**PinX1 inhibits migration and invasion of breast cancer cells by suppressing MMP-9 expression and activity. (A)** Western blot analysis of the relative protein levels of PinX1, MMP-2, MMP-9, TIMP-1 and TIMP-2 in PinX1 siRNA and control siRNA group for both MDAMB-231 and BT-549 cell lines. **(B)** Western blot analysis of the relative protein levels of PinX1, MMP-2, MMP-9, TIMP-1 and TIMP-2 in PinX1^OE^ and Vector groups for both MDA-MB-231 and BT-549 cell lines. **(C)** Top panel, gelatin zymography analysis of the relative enzyme activities of MMP-9 in PinX1 knockdown and control siRNA group for both MDA-MB-231 and BT-549 cell lines. Bottom panel, gelatin zymography analysis of the relative enzyme activities of MMP-9 in PinX1^OE^ and Vector groups for both MDA-MB-231 and BT-549 cell lines. **(D)** Western blotting of PinX1 and MMP-9 in there groups of control siRNA, PinX1 siRNA, and PinX1 siRNA co-treated with MMP-9 inhibitor I for both MDA-MB-231 and BT-549 cell lines. **(E)** Gelatin zymography analysis of MMP-9 in there groups of control siRNA, PinX1 siRNA, and PinX1 siRNA co-treated with MMP-9 inhibitor I for both MDA-MB-231 and BT-549 cell lines. **(F)** and **(G)** The increased abilities of migration and invasion regulated by PinX1 knockdown in breast cancer cells was blocked by MMP-9 inhibitor I. All experiments were carried out in triplicate. Histograms represent means ± SD. ***, *P* < 0.001.

To investigate the mechanisms of PinX1 regulating migration and invasion in breast cancer cells, we performed western blot to detect the MMPs protein levels and gelatin zymography to observe the MMPs activity. Our result showed that the MMP-9 expression and activity were negatively regulated by PinX1 in MDA-MB-231 and BT-549 cells,but not MMP-2 (Figure 3A & B & C). So we supposed PinX1 suppress migration and invasion of breast cancer cells by regulating MMP-9 expression and activity. To further validate our assumption, we added MMP-9 inhibitor I (sc-311437, Santa Cruz) at the same time of PinX1 siRNA transfecting into cells. As expected, the upregulation of MMP-9 was blocked by MMP-9 inhibitor I (Figure 3D & E). We also validated this hypothesis by migration and invasion analysis, the migration and invasion ability can be enhanced by knocking down PinX1 in MDA-MB-231 and BT-549 cells, nevertheless, these regulations were blocked by MMP-9 inhibitor I (Figure 3F & G).

As we know TIMP-1 and TIMP-2 is the tissue inhibitor of MMP-9 and MMP-2, so we detected the expression of TIMP protein. Our data showed that MMP-9 expression was up-regulated or down-regulated corresponded with PinX1 knockdown or overexpression, however, the expression of TIMP-1 had not changed correspondingly. Moreover, neither overexpressing nor silencing PinX1 had any effect on the protein levels of MMP-2 and TIMP-2 (Figure 3A & B).

### PinX1 suppress MMP-9 expression via NF-κB-dependent transcription

**Figure 4:**
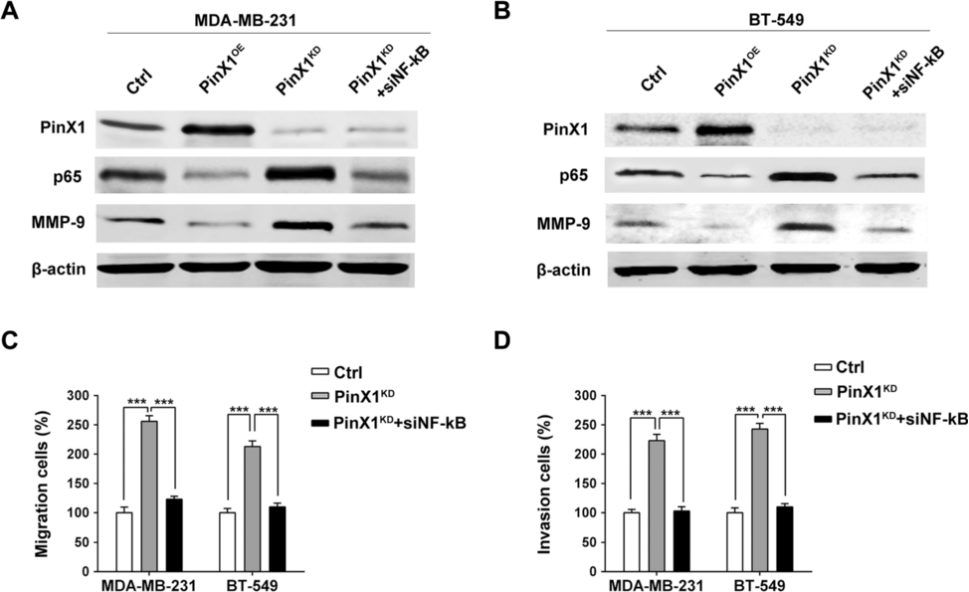
**PinX1 suppress MMP-9 expression via NF-κB-dependent transcription. (A)** and **(B)** Western blot analysis of the relative protein levels of PinX1, MMP-9 and NF-κB-p65 in four groups of control, PinX1^OE^ , PinX1^KD^ and PinX1^KD^ co-treated with NF-κB-p65 siRNA for both MDA-MB-231 and BT-549 stable cell lines. The NF-κB specific siRNA dramatically prevented the up-regulation of MMP-9 expression induced by PinX1 knockdown. **(C)** and **(D)** The increased ability of migration and invasion regulated by PinX1 knockdown was suppressed by NF-κB-p65 siRNA in both MDA-MB-231 and BT-549 stable cell lines. All experiments were carried out in triplicate. Histograms represent means ± SD. ***, *P* < 0.001.

Furthermore, increasing evidence demonstrate that several MMPs (including MMP-9) expression and activation were regulated by NF-κB activation in many human cancers [27,28]. G-patch domain of PinX1 is also an important nucleic acids binding domain which could combine with the C-terminus of the NF-κB-repression factor (NRF) [29]. Thus, PinX1 may also inhibit the transcriptional activity of NF-κB proteins by direct protein-protein interaction with its G-patch domain. Western blot results showed that the level of NF-κB-p65 protein was dramatically increased in PinX1^KD^-MDA-MB-231 cells and PinX1^KD^-BT-549 cells. In contrast, NF-κB-p65 expression was down-regulated sharply in PinX1^OE^-MDA-MB-231 cells and PinX1^OE^-BT-549 cells (Figure 4A & B). To further confirm whether PinX1 regulated MMP-9 expression via the NF-κB signaling pathway in human breast cancer cells, we transfected NF-κB-p65 siRNA (Santa Cruz) into PinX1^KD^-MDA-MB-231 cells and PinX1^KD^-BT-549 cells. Our result indicated that the MMP-9 expression was up-regulated in PinX1^KD^-MDA-MB-231 cells and PinX1^KD^-BT-549 cells, but these effects were further blocked by silencing NF-κB-p65 with the specific siRNA (Figure 4A & B). These data provide definite evidence that PinX1 may modulate MMP-9 expression by NF-κB transcription factor.

We also validated the mechanism by migration and invasion analysis. The migration and invasion ability can be enhanced in PinX1^KD^-MDA-MB-231 cells and PinX1^KD^-BT-549 cells, however, these effects were dramatically reversed by treatment with NF-κB-p65 siRNA (Figure 4C & D). These data suggested that PinX1 may regulate the migration and invasion via the NF-κB/MMP-9 signaling pathway.

### PinX1 inhibits breast cancer cells metastasis in vivo

**Figure 5:**
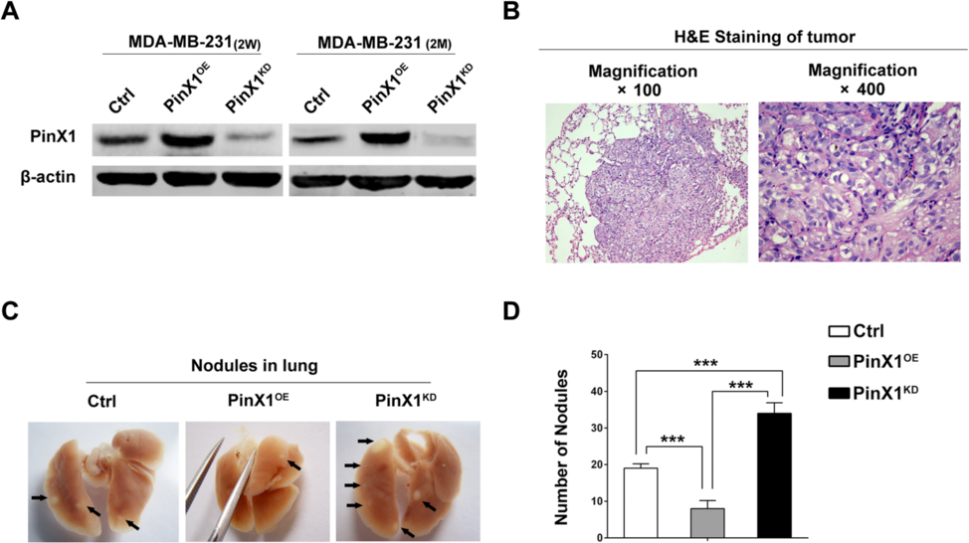
**PinX1 inhibits breast cancer cells metastasis in vivo. (A)** Left panel, Western blotting of PinX1 in there groups of Ctrl-MDA-MB-231 cell lines, PinX1^OE^-MDA-MB-231 cell lines and PinX1^KD^-MDA-MB-231 cell lines, which was selected with puromycin for 2 weeks after lentivirus infection. Right panel, without puromycin selection for 2 months, PinX1 expression levels were not changed in MDA-MB-231 stable cell lines. **(B)** H&E staining sections of lungs 2 months after injection of PinX1^KD^-MDA-MB-231 cell lines in BALB/c nude mouse through tail vein. Left panel, magnification × 100; right panel, magnification × 400. **(C)** Representative images of 10% buffered formalin fixed lungs with metastatic nodules 2 months after injection of Ctrl, PinX1^OE^ and PinX1^KD^ MDA-MB-231 stable cell lines. Arrows indicate metastatic nodules. **(D)** The number of lung metastatic nodules was counted under a dissecting microscope. Compared with the PinX1^OE^ group, a statistically dramatic increase in the number of the lung metastases was seen in PinX1^KD^ group, and these two groups also had significant diversity compared with Ctrl group respectively. Data are displayed with means ± SD from 10 mice in each group. ***, *P* < 0.001.

Lastly, we examined whether PinX1 suppressed breast cancer metastasis in vivo. PinX1^OE^-MDA-MB-231, PinX1^KD^-MDA-MB-231 and Ctrl-MDA-MB-231 cell lines were established previously. After 3 weeks selection following with lentivirus infection, the PinX1 protein levels of these cell lines were confirmed by western blot. Then continuing to incubate them without adding puromycin for 2 months, we determined that the PinX1 protein expression levels of three stable cell lines had not been changed (Figure 5A).

The BALB/c nude mice were randomly divided into three groups: PinX1^OE^, PinX1^KD^ and Control group, each group consisted of 10 mice. Three groups of nude mice were injected through tail vein with PinX1^OE^-MDA-MB-231, PinX1^KD^-MDA-MB-231 and Ctrl-MDA-MB-231 cells respectively. After 2 months, three groups of mice were sacrificed and their lungs were resected and fixed in 10% buffered formalin for metastatic nodules counting and further histopathological analysis. Randomly selected metastatic nodules had been validated by H&E staining (Figure 5B). Extensive tumor formation was found in PinX1^KD^ group. In contrast, the lungs in PinX1^OE^ group had fewer and smaller detectable tumor nodules (Figure 5C). Compared with PinX1^OE^ group, the dramatic increase in the number of the tumor nodules was observed in PinX1^KD^ group, meanwhile, PinX1^OE^ group and PinX1^KD^ group had significant differences compared with Control group respectively (Figure 5D).

## Discussion

PinX1 is a functional gene at human chromosome 8p23, a region frequently associated with loss of heterozygosity (LOH) in a variety of human malignancies [13]. Increasing evidence demonstrate that PinX1 plays a key role as a putative tumor suppressor in human cancer progression [18,19]. However, the expression and function of PinX1 in breast cancer and its correlation with the clinicopathological features of breast cancer patients have never been investigated.

In the present study, we used a breast cancer TMA containing 405 tumor samples with specific clinical data to investigate the role of PinX1 in human breast cancer. Our data showed that low PinX1 expression was associated with lymph node metastasis and histology grade in patients, as well as with poorer overall and disease-specific survival (Figure 1B & C; Table 1). Cox regression analysis revealed that low PinX1 expression was an independent negative prognostic indicator for breast cancer patients (Table **2**; Table 3). These findings suggested a potential role of PinX1 in regulating breast cancer metastasis and functioning as a breast cancer candidate clinical prognostic marker. Our clinical data urged us to carry out a series of in vitro and in vivo experiments to explore the potential mechanisms.

The matrix metalloproteinase (MMP) family can degrade the extracellular matrix (ECM) in the major early stages of a number of malignant tumors, which plays an important role in cancer invasion and metastasis [30]. High expression of MMP-9 and MMP-2 were associated with lymph node metastasis as well as with poorer survival in breast cancer [31]. Our data demonstrated that PinX1 inhibited breast cancer cells’ migration and invasion abilities by down-regulating MMP-9 expression and activity in vitro (Figure 2; Figure 3). MMPs activity is controlled by specific, endogenous tissue inhibitors of metalloproteinases (TIMPs), and the imbalance between MMPs and TIMPs is responsible for cancer metastasis [32]. TIMP-1 is the tissue inhibitor of MMP-9, which negatively regulating MMP-9 enzyme activity were involved in several tumor metastasis processes, including breast cancer [33]. But in our study, TIMP-1 expression was not seemed to be the regulator of MMP-9 activation in breast cancer cells (Figure 3A & B). These result suggested us to investigate the potential mechanism of how PinX1 regulates MMP-9 expression and activity.

NF-κB is a critical transcription factor activated in various types of human cancers and plays a crucial role in tumor development and progression [34,35]. The NF-κB signaling modulates several key biological processes during the development and progression of cancer by inducing transcription of a variety of target genes that regulate cell proliferation, survival, invasion and angiogenesis [36-38]. NF-κB is constitutively present in cells as a heterodimer, consisting of a p50 DNA-binding subunit and a p65 transactivating subunit [39]. Furthermore, increasing evidence demonstrate that MMP-9 expression and activation were regulated by p65 up-regulation and nuclear translocation which induced NF-κB activation in many human cancers [27,28]. Our data indicated that the NF-κB specific siRNA dramatically prevented the up-regulation of MMP-9 expression induced by PinX1 knockdown (Figure 4A & B). Consistently, the increased ability of migration and invasion induced by PinX1 knockdown was also suppressed by inhibition of NF-κB-p65 expression in breast cancer cells (Figure 4C & D). Thus, these results suggested that PinX1 may regulate the migration and invasion via the NF-κB/MMP-9 signaling pathway.

**Figure 6:**
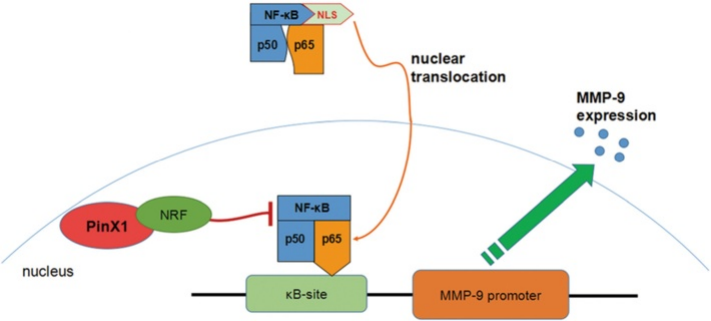
**A Model of PinX1 suppresses MMP-9 expression via NF-κB signaling pathway.** We speculate that PinX1 can suppress the expression of MMP-9 owing to the inhibition of transcriptional activity of NF-κB p65 proteins by direct protein-protein interaction with its G-patch domain which could combine with the C-terminus of the NF-κB-repression factor (NRF).

We regret to investigate the potential regulation mechanism between PinX1 and NF-κB pathway, however, we have found some conceivable relations between them. Human PinX1 protein contains an N-terminal Gly-rich patch (G-patch) and a C-terminal TID (telomerase inhibitory domain) [12]. G-patch domain existed in a number of putative RNA-binding proteins involved in tumor suppression and DNA-damage repair [40], but the function of them had been researched rarely. Moreover, G-patch domain of PinX1 is also an important nucleic acids binding domain which could combine with the C-terminus of the NF-κB-repression factor (NRF) [29]. NRF is a nuclear inhibitor of NF-κB, which can inhibit the transcriptional activity of NF-κB proteins by direct protein-protein interaction [41]. Thus, we conclude that PinX1 also can inhibit the transcriptional activity of NF-κB proteins by direct protein-protein interaction with its G-patch domain (Figure 6). These hypothesizes have never been definitely validated. However, the association between PinX1 and NF-κB in breast cancer cells exists indeed. So we will investigate the further molecular mechanisms between PinX1 and NF-κB signaling pathway continually.

To further observe the functional role of PinX1 in breast cancer metastasis in vivo, three groups of nude mice experimental model were constructed. We investigated that PinX1 overexpression in breast cancer cells significantly inhibited the formation of metastasis nodules in lung of nude mice. In contrast, PinX1 knockdown dramatically enhanced the metastasis process (Figure 5).

In conclusion, loss of PinX1 expression was significantly correlated with breast cancer progression and was an independent negative prognostic factor in breast cancer patients. PinX1 suppressed breast cancer migration and invasion by inhibiting the expression and activity of MMP-9 via NF-κB-dependent transcription. Therefore PinX1 may be an attractive therapeutic target for the treatment of breast cancer.

## Materials and methods

### Patient specimens and tissue microarray construction

The collection of patient specimens and construction of the tissue microarray (TMA) have been previously described [42]. The study material consists of a series of 405 consecutive cases of primary breast carcinoma, from The First Affiliated Hospital of Nanjing Medical University, between 1996 and 2005. All these patients were treated with surgery only or with postoperative adjuvant therapy. The patients’ clinicopathologic information including age at diagnosis, tumor size, histology grade, lymph node metastasis, ER status, PR status, HER2 status and p53 status was obtained from the archive of the pathology department and confirmed by the medical record of the hospital. The histologic grade was assessed using Bloom-Richardson classification. Five-year clinical follow-up results were available for 236 patients. The use of these tissue specimens was approved by the Ethics Committee of the Hospital.

### Immunohistochemistry of TMA

Immunohistochemistry was carried out as described before [43]. According to the streptavidin-peroxidase (Sp) method using a standard Sp Kit (Zhongshan biotech, Beijing, China). The TMA slides were dewaxed at 60°C for 20 min followed by three 5-min washes with xylene. The tissues were then rehydrated by washing the slides for 5-min each with 100%, 95%, 80% ethanol and finally with distilled water. The slides were then heated to 95°C for 30 min in 10 mmol/L sodium citrate (pH 6.0) for antigen retrieval and then treated with 3% hydrogen peroxide for 1 hour to block the endogenous peroxidase activity. Then subsequently the TMA slides were incubated with a polyclonal rabbit anti-PinX1 antibody (1:50 dilution; Novus Biologicals, Littleton, CO, USA) at 4°C overnight, and 3, 3′-diaminobenzidine (DAB; Zhongshan Biotech, Beijing, China) was used to produce a brown precipitate. Negative controls were performed by substituting primary antibodies with non-immune serum.

### Evaluation of immunostaining

The evaluation of PinX1 staining was blindly and independently examined by two pathologists. Positive PinX1 immunostaining is defined mainly in the nucleus area and also can be observed in the cytoplasm. We grade it according to both the intensity and percentage of cells with positive staining. The PinX1 staining intensity was scored 0 to 3 (0 = negative; 1 = weak; 2 = moderate; 3 = strong). The percentage of PinX1-positive stained cells was also scored into four categories: 1 (0%-25%), 2 (26%-50%), 3 (51%-75%) and 4 (76%-100%). The level of PinX1 staining was evaluated by the immunoreactive score (IRS), which is calculated by multiplying the scores of staining intensity and the percentage of positive cells. Based on the IRS, the PinX1 staining pattern was categorized as negative (IRS: 0), weak (IRS: 1–3), moderate (IRS: 4–6) and strong (IRS: 8–12). The concordance for IRS staining score of PinX1 between the two pathologists was 363 in 405 samples (90%), and the few discrepancies were resolved by consensus using a multihead microscope.

The optimum cutoff value of IRS was obtained by receiver-operator characteristic analysis, and the areas under the curves at different cutoff values of the PinX1 IRS for 1, 3 and 5 years of overall survival time were also calculated. The optimum value of cutoff point of the PinX1 IRS was shown to be 3 since it had the best predictive value for survival (Additional file 1: Figure S1). Under these conditions, samples with IRS 0–3 and 4–12 were classified as having low or high expression of PinX1, respectively.

### Animals and cell lines

Female BALB/c nude mice, 6 weeks old, were purchased from the Shanghai Laboratory Animal Center (Shanghai, China) for studies approved by the Animal Care Committee of Xuzhou Medical College. Two human breast cancer cell lines MDA-MB-231 and BT-549 were purchased from the Shanghai Institute of Biochemistry and Cell Biology, Chinese Academy of Sciences (Shanghai, China). MDA-MB-231 cells were cultured in Leibovitz’ s L-15 Medium (Gibco, USA) supplemented with 10% fetal calf serum (Gibco, USA), BT-549 cells were cultured in RPMI 1640 Medium (Invitrogen, Shanghai, China) supplemented with 10% fetal calf serum. These two cells were both incubated in a 37°C humidified incubator with 5% CO_2_.

### siRNA and DNA transfections, and stable cell line generation

Cells were grown to 50% confluence before small interfering RNA (siRNA) transfection. PinX1 siRNA (GenePharma, Shanghai, China), NF-κB-p65 siRNA (Santa Cruz, CA, USA) or Nonspecific control siRNA (GenePharma, Shanghai, China) was transfected by siLentFect Lipid Reagent (Bio-Rad, CA, USA) according to the manufacturer’s protocol. The pEGFP-C3 and pEGFP-C3-PinX1 expression plasmids were obtained from Dr Xiao-Fen Lai (Southern Medical University, Guangzhou, China). Transfection of the pEGFP-C3-PinX1 plasmid and the pEGFP-C3 vector into the MDA-MB-231 and BT-549 cells were carried out using Lipofectamine 2000 transfection reagent (Invitrogen) following the manufacturer’s instructions.

PinX1 overexpression MDA-MB-231 cell lines (PinX1^OE^-MDA-MB-231), PinX1 knockdown MDA-MB-231 cell lines (PinX1^KD^-MDA-MB-231) and control MDA-MB-231 cell lines (Ctrl-MDA-MB-231) were established by infecting with lentivirus packing PinX1 expression vector, PinX1 shRNA expression vector and control vector respectively (GenePharma, Shanghai, China). It is the same as the construction of PinX1 overexpression BT-549 cell lines (PinX1^OE^-BT-549), PinX1 knockdown BT-549 cell lines (PinX1^KD^-BT-549) and control BT-549 cell lines (Ctrl-BT-549). Target cells were infected with lentivirus for 48 hours then selected with puromycin (Santa Cruz) for three weeks.

### Western blot analysis

Western blot analysis was performed as described previousely [44]. The following antibodies were used for western blot: rabbit anti-PinX1 (Novus Biologicals); rabbit anti-MMP-2, anti-MMP-9, anti-TIMP-1, anti-TIMP-2 (all from Cell Signaling Technology); rabbit anti-NF-κB-p65 (Santa Cruz Biotechnology); mouse anti-β-actin (Cell Signaling Technology); Infrared IRDye-labeled secondary antibody (LI-COR, NE, USA) was applied to the blot for 2 hour at room temperature, the signals were detected with Odyssey Infrared Imaging system (LI-COR).

### Cell migration and invasion assay

Cell migration and invasion assay were performed using modified two-chamber plates with a pore size of 8 μm. The transwell filter coating with or without Matrigel (BD Biosciences, Mississauga, Canada) were used respectively for invasion and migration assay. The detailed conditions were described previously [45].

### Gelatin zymography

Gelatin zymography was performed as described before [46]. Thirty-six hours after transfection, cells were incubated in serum-free medium for 24 h. The proteins in the conditioned medium were concentrated with Amicon Ultra-4-30 k centrifugal filters (Millipore, MA, USA) at 7500 g for 20 min at 4°C. Fifty micrograms of the proteins were loaded on a 10% polyacrylamide gel containing 0.1% gelatin (Sigma, MO, USA). Gelatinolytic activity was shown as clear areas in the gel. Gels were photographed and then quantitatively measured by scanning densitometry.

### Tail vein assay of metastasis

The BALB/c nude mice were randomly divided into three groups: PinX1^OE^, PinX1^KD^ and Control group, each group consisted of 10 mice. The mice were injected intravenously with 2.5 × 10^6^ MDA-MB-231 cells in 200 μl of PBS through tail vein. After 2 months, the three groups of mice were sacrificed, their lungs were resected and fixed in 10% buffered formalin for metastatic nodules counting and further histopathological analysis. The number of metastatic nodules presented on the surface of each group of lungs was counted by visual inspection using a stereoscopic dissecting microscope.

### Statistical analysis

For the TMA, statistical analysis was performed with SPSS 20 software (SPSS, Inc, Chicago, IL). The association between PinX1 staining and the clinicopathologic parameters of the breast cancer patients was evaluated by χ^2^ test. The Kaplan-Meier method and log-rank test were used to evaluate the correlation between PinX1 expression and patient survival. Cox regression model was used for multivariate analysis. Data are expressed as the means ± SD. Two-factor analysis of variance procedures and the Dunnett’s t-test were used to assess differences within treatment groups. Differences were considered significant when *P* < 0.05.

## Additional file

Additional file 1: Figure S1. Receiver operating characteristic (ROC) curve is obtained to determine the optimal cutoff value of PinX1 expression. ROC obtains the area under the curves (AUCs) at different cutoff values of PinX1 immunoreactivity score (IRS) for 1, 3 and 5 years of overall survival time.

## Abbreviations

PinX1: PIN2/TRF1-interacting telomerase inhibitor 1
hTERT: Human telomerase reverse transcriptase
TRF1: Telomeric repeat binding factor 1
LOH: Loss of heterozygosity
MMP: Matrix metalloproteinase
TIMPs: The specific, endogenous tissue inhibitors of metalloproteinases
TID: Telomerase inhibitory domain
NRF: The NF-κB-repression factor
ECM: The extracellular matrix
TMA: The tissue microarray
IRS: The immunoreactive score
