# Supplementary material for: PinX1 inhibits the invasion and metastasis of human breast cancer via suppressing NF-κB/MMP-9 signaling pathway
Source: Mol Cancer. 2015 Mar 26;14:66. doi: 10.1186/s12943-015-0332-2 (PMC4404090; doi:10.1186/s12943-015-0332-2)

**Supplementary Figure 1 Receiver operating characteristic (ROC) curve is obtained to determine the optimal cutoff value of PinX1 expression**. ROC obtains the area under the curves (AUCs) at different cutoff values of PinX1 immunoreactivity score (IRS) for 1, 3 and 5 years of overall survival time.


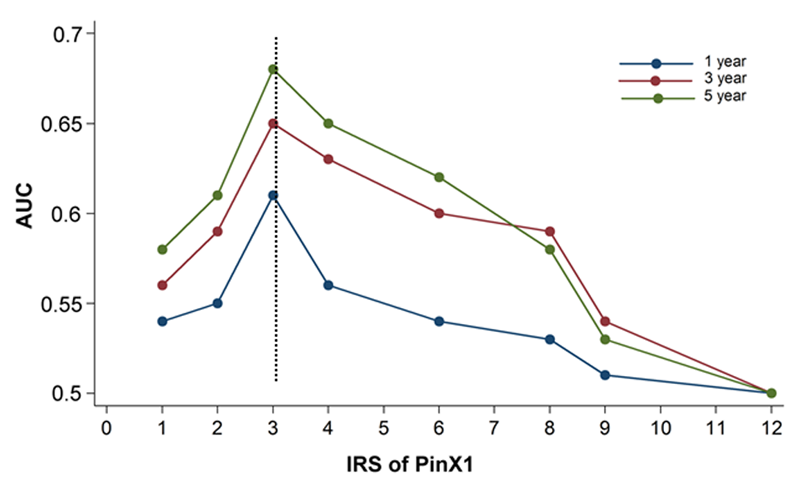

Supplement: Additional file 1: Figure S1. — Receiver operating characteristic (ROC) curve is obtained to determine the optimal cutoff value of PinX1 expression. ROC obtains the area under the curves (AUCs) at different cutoff values of PinX1 immunoreactivity score (IRS) for 1, 3 and 5 years of overall survival time. [file 12943_2015_332_MOESM1_ESM.docx]
